# Competing risks and cancer-specific mortality: why it matters

**DOI:** 10.18632/oncotarget.23729

**Published:** 2017-12-28

**Authors:** Kay See Tan, Takashi Eguchi, Prasad S. Adusumilli

**Affiliations:** Prasad S. Adusumilli: Memorial Sloan Kettering Cancer Center, New York, NY, USA; Center for Cell Engineering, Memorial Sloan Kettering Cancer Center, New York, NY, USA

**Keywords:** competing risks, lung cancer, Kaplan-Meier, survival, cumulative incidence

In studies with multiple endpoints, such as cancer-specific mortality and noncancer-specific mortality, conventional statistical approaches evaluate these endpoints using a separate Kaplan-Meier analysis without considering whether these endpoints are competing events for each other.

In our recent study, we examined the influence of increasing age on lung cancer-specific and noncancer-specific mortality among patients with early-stage, non-small cell lung cancer (NSCLC) using a competing risk analysis [1]. We found that both the 5-year lung cancer-specific and noncancer-specific cumulative incidence of death increase with age. We also reported a higher incidence of noncancer-specific mortality compared with lung cancer-specific mortality among patients who were ≥75 years of age that lasted up to 2 years postresection. These findings highlight the necessity of accounting for noncancer-specific mortality as a competing event when assessing cancer-specific mortality in elderly patients. Our article sheds light on two related concepts—competing risks and the statistical challenges that arise from competing risks in the elderly patient population.

Clinical research that involves time-to-event endpoints, such as all-cause mortality, conventionally uses the Kaplan-Meier approach that includes censoring patients at the end of the follow-up period. If the event of interest is cancer-specific mortality, a patient may die due to causes unrelated to the disease; such events are termed “competing risk events.” Clinical research also may focus on non-mortality related outcomes such as incidence of disease recurrence, second primary cancer, and treatment success. In these incidences, death without observation of the event of interest will be considered a competing risk event. Competing risk events may substantially alter the probability of occurrence of the event of interest or even preclude its onset. Statistical methods have been developed to assess time-to-event outcomes in the presence of competing risks (competing risk approaches).

With a growing elderly population, the recognition and proper examination of competing risks within the intersection of geriatric and oncologic research is more important than ever. A higher proportion of elderly patients consequently increases the incidence of diseases that are attributable to aging and frailty, thus making the cohort of elderly patients highly susceptible to competing risk events. A review of 50 clinical studies published in high-impact journals that were focused on the aging or multimorbid population found competing risk issues in 70% of those studies [2].

The conventional Kaplan-Meier framework assumes that censoring is uninformative since, without competing risk events, the estimates from both the naïve Kaplan-Meier and competing risk approaches are identical. However, the presence of competing risk events induces informative censoring that produces estimates of incidence that are biased upwards, even under the untestable assumption that the competing events are independent of one another [3].

The resulting probability of the event of interest over time is generally overestimated, which leads to biased findings and inaccurate measurements of clinical effectiveness (e.g., number needed to treat) [4]. A recent review of 100 studies from prominent medical journals found that 46% of studies that used Kaplan-Meier estimates ignored potential competing risks and Kaplan-Meier estimates were biased by at least 10% [5]. Additionally, a disregard for competing risks implies that Kaplan-Meier estimates should be interpreted only within the hypothetical population; typically, this is not the appropriate population of clinical interest as competing risks do not exist.

**Figure 1:**
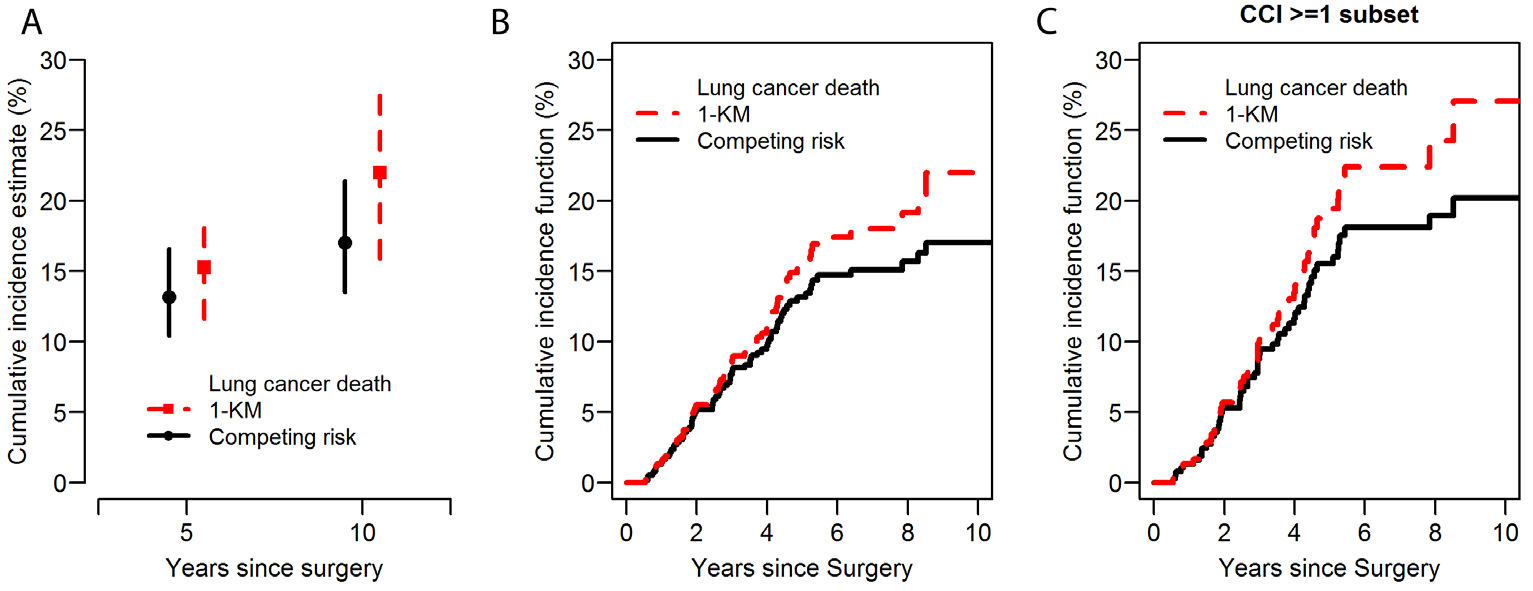
Lung cancer-specific cumulative incidence of death (CID) curves: a comparison between 1-Kaplan-Meier approach and the competing risk approach **A.** 5-year and 10-year lung cancer–specific cumulative incidence of death with a 95% confidence interval. Red square dot: 5-year and 10-year estimated lung cancer–specific CID based on 1-Kaplan-Meier approach with one endpoint and censorship of competing risks. Black circle dot: 5-year and 10-year estimated lung cancer–specific CID based on competing risk analysis in which noncancer–specific death are taken into account as a competing risk event. Red dashed line and black solid line represent 95% confidence intervals. **B.** Lung cancer–specific CID curves in all patients. Red dashed line: the classic 1-Kaplan-Meier curves with one endpoint and censorship of competing risks. Black solid line: cumulative incidence curve that takes into account non-cancer specific death as a competing risk event. **C.** Lung cancer–specific CID curves in patients with comorbidities (Charlson comorbidity index ≥ 1). CCI, Charlson comorbidity index; CID, cumulative incidence of death; KM, Kaplan-Meier.

Among solid tumors, lung cancer carries a relatively high risk of competing cancer and noncancer events since more than two-thirds of lung cancer patients are ≥65 years of age at time of diagnosis, half of whom are ≥75 years of age [6]. It has been reported that noncancer risk factors, such as increased age, comorbidity (e.g., chronic obstructive pulmonary disease and cardiovascular disease), and poor pulmonary function, influence outcomes of patients with NSCLC [7]. To illustrate the distinction between findings, both accounting for and not accounting for competing risks, we present the cumulative incidence curves of cancer-specific death (Figure 1) using data from our recent study [1]. In this example, we focused on 638 patients who were ≥75 years of age, among which 63% had at least one major comorbidity (Charlson comorbidity index ≥1) at baseline. We present the cumulative incidence estimates, which are based on the classic Kaplan-Meier approach (1-Kaplan-Meier) as opposed to the competing risk approach, in the overall cohort. In this context, deaths from noncancer-specific causes are considered competing events. The naïve Kaplan-Meier approach—where noncancer-specific death are censored—overestimates the cumulative incidence of cancer-specific death compared with the curves that take competing risks into account; this is particularly true as follow-up continues beyond the 5-year period (Figure 1A & 1B). The magnitude of overestimation using the Kaplan-Meier approach is even greater among patients with comorbidities (Figure 1C), who are a subset of patients with higher incidence of noncancer-specific deaths. This overestimation is due to the inappropriate assumption regarding censoring during the analysis. In the Kaplan-Meier analysis, the patients who died of noncancer-specific causes (competing risk event) are treated as censored and assumed to have the same chance of lung cancer death (event of interest) after the same period of time as the patients who continued to be followed. As a result, the probability of the event of interest is overestimated.

In summary, the ubiquity of competing risks in medical research and their potential to bias research findings necessitates careful consideration of competing events when estimating disease incidence. In these circumstances, particularly among study populations who are susceptible to competing events such as geriatric or critically-ill patients, we recommend a competing risk approach. For technical details and implementation of competing risk analysis, we direct readers to the tutorial paper by Putter et al [8]. This competing risk approach will result in more accurate estimates of disease incidence and better determination of patient risk that will be used during the clinical decision-making process.
